# Thermal Stability and Flame Retardancy of Polypropylene Composites Containing Siloxane-Silsesquioxane Resins

**DOI:** 10.3390/polym10091019

**Published:** 2018-09-13

**Authors:** Arkadiusz Niemczyk, Katarzyna Dziubek, Beata Sacher-Majewska, Krystyna Czaja, Justyna Czech-Polak, Rafał Oliwa, Joanna Lenża, Mariusz Szołyga

**Affiliations:** 1 Faculty of Chemistry, University of Opole, Oleska 48, 45-052 Opole, Poland; katarzyna.dziubek@uni.opole.pl (K.D.); beata.sacher@uni.opole.pl (B.S.-M.); krystyna.czaja@uni.opole.pl (K.C.); 2 Faculty of Chemistry, Rzeszow University of Technology, Al. Powstańców Warszawy 6, 35-959 Rzeszów, Poland; j.czech@prz.edu.pl (J.C.-P.); oliwa@prz.edu.pl (R.O.); 3 Central Mining Institute, Plac Gwarków 1, 40–166 Katowice, Poland; jlenza@gig.eu; 4 Centre for Advanced Technologies, Adam Mickiewicz University in Poznań, Umultowska 89C, 61-614 Poznań, Poland; markussqueeze@wp.pl

**Keywords:** siloxane-silsesquioxane resins, polypropylene, thermogravimetry, cone calorimeter tests, flame-retardant mechanism

## Abstract

A novel group of silsesquioxane derivatives, which are siloxane-silsesquioxane resins (S4SQ), was for the first time examined as possible flame retardants in polypropylene (PP) materials. Thermal stability of the PP/S4SQ composites compared to the S4SQ resins and neat PP was estimated using thermogravimetric (TG) analysis under nitrogen and in air atmosphere. The effects of the non-functionalized and *n*-alkyl-functionalized siloxane-silsesquioxane resins on thermostability and flame retardancy of PP materials were also evaluated by thermogravimetry-Fourier transform infrared spectrometry (TG-FTIR) and by cone calorimeter tests. The results revealed that the functionalized S4SQ resins may form a continuous ceramic layer on the material surface during its combustion, which improves both thermal stability and flame retardancy of the PP materials. This beneficial effect was observed especially when small amounts of the S4SQ fillers were applied. The performed analyses allowed us to propose a possible mechanism for the degradation of the siloxane-silsesquioxane resins, as well as to explain their possible role during the combustion of the PP/S4SQ composites.

## 1. Introduction

Polymers (in particular polyolefins) have become an important class of materials in recent years, mostly due to their favorable properties, relatively low cost and easy accessibility. However, the insufficient thermal stability and flammability of these materials still constitute a serious problem which may limit their potential multidirectional applications [1]. Moreover, well-known and effective halogenated flame retardants have been gradually prohibited as they are environmentally toxic [2]. Thus, special efforts are being made to develop a new class of flame retardants for polymeric materials as an alternative for the commonly used halogenated additives. Many studies have already demonstrated that silicones, silanes, silsesquioxanes, silicas and silicates may improve the thermal stability of polymeric materials and that they could be explored as potential environmentally friendly flame retardants [3,4,5,6].

Among silicon-based compounds, special attention should be paid to polyhedral oligomeric silsesquioxanes (POSS), which have been successfully applied as fillers for various polymers [7,8,9,10,11,12,13,14], including polyolefins [15,16,17,18,19,20,21,22,23,24,25]. The presence of POSS molecules in these materials contributes generally to improved mechanical [18,24,26,27,28] and rheological [22,29,30,31] properties, as well as influenced their melting and crystallization behavior [26,29,31,32,33,34]. Moreover, it has already been demonstrated that silsesquioxanes may significantly improve thermal and thermo-oxidative stability [16,18,21,28,30,32,34,35,36,37], as well as reduce flammability of polyolefin-based composites [4,28,33]. However, despite the obvious advantages of POSS compounds, their potential commercial applications are limited as they are relatively expensive [38,39,40].

The search for low-cost alternatives to POSS molecules led to a new class of hybrid silicon compounds: siloxane-silsesquioxane resins [41,42,43,44,45,46,47,48,49,50,51,52,53]. They have extended network structures which are composed of silsesquioxane cages connected by siloxane chains that can be functionalized, and which affect their unique properties [51,52,53].

However, there are only a few papers available to date which report on the application of siloxane-silsesquioxane resins as fillers [38,39,40,54]. The influence of non-functionalized and *n*-alkyl-functionalized siloxane-silsesquioxane resins on the morphological, structural, thermal and mechanical properties of polypropylene composites was presented in our previous work [54]. It was demonstrated that the presence itself and the length of the alkyl groups in the resin structures play a crucial role in the dispersion of the filler particles in the polypropylene (PP) matrix. The applied resins affected the crystalline structure and improved the crystallization behavior of the polymer matrix. Moreover, the presence of the siloxane-silsesquioxane resin particles enhanced the impact strength and elongation at break of the composites [55]. Subsequently, phenyl-functionalized siloxane-silsesquioxane resin (SiOPh) was applied by Dobrzyńska-Mizera et al. to control the nucleation efficiency of polypropylene composites containing sorbitol derivatives [38,39,40]. It should, however, be emphasized that there are no literature data available on the thermal stability and fire behavior of polymer composites containing siloxane-silsesquioxane resins as fillers.

In this article, the influence of siloxane-silsesquioxane resins on thermal stability and fire behavior of the polypropylene matrix is discussed. Composites containing 1–10 wt % of non-functionalized (S4SQ-H) as well as *n*-octyl- and *n*-octadecyl-functionalized resins (S4SQ-8 and S4SQ-18, respectively) were prepared by the melt blending method. The thermal stability of neat siloxane-silsesquioxane resins, as well as of the obtained composites was examined by the thermogravimetric (TG) analysis in both inert and oxidative environments. The selected materials were also studied by thermogravimetry-Fourier transform infrared spectrometry (TG-FTIR). Finally, the flame retardancy of the PP composites filled with siloxane-silsesquioxane resins was evaluated on the basis of cone calorimeter tests.

## 2. Materials and Methods

### 2.1. Materials

Polypropylene (PP) Moplen HP 400 R (MFR = 23 g/10 min) was provided by Basell Orlen Polyolefins, and was used as the polymer matrix. The non-functionalized (S4SQ-H) and *n*-alkyl-functionalized (S4SQ-8 and S4SQ-18) siloxane-silsesquioxane resins were synthesized in the Centre for Advanced Technologies of the Adam Mickiewicz University in Poznań, according to procedures described elsewhere [51,52,53,54]. The S4SQ designation identifies a siloxane-silsesquioxane resin composed of the silsesquioxane cages connected by 4 siloxane groups [54].

### 2.2. Composites Preparation

Polypropylene composites filled with siloxane-silsesquioxane resins (S4SQ) were prepared by the melt blending method. In the first step, PP masterbatches containing 20 wt % of S4SQ resins were prepared using a HAAKE PolyLab Rheomether (Thermo Fisher Scientific, San Jose, CA, USA) (180 °C, 50 rpm, 15 min). In the second step, the obtained masterbatches were blended with neat PP granulate at suitable weight ratios. The PP/S4SQ composites were obtained with a IM-15 laboratory conical twin screw extruder (ZAMAK, Skawina, Poland) (175–195 °C, 100–200 rpm) coupled with a IMM-15 laboratory injection machine (ZAMAK, Skawina, Poland) (190 °C, 6 MPa). The obtained composites contained 1, 5 and 10 wt % of the appropriate S4SQ resins.

### 2.3. Characterization

The thermogravimetric analysis of neat siloxane-silsesquioxane resins was carried out using a Q50-TGA thermogravimeter (TA Instruments, Inc., New Castle, DE, USA) under the flow of N_2_ or air. The samples (15–25 mg) were loaded on platinum pans and heated from room temperature to 1000 °C at the rate of 10 °C/min. The thermal stability of the obtained polymer materials was evaluated by thermogravimetric (TG) analysis using a TG/DSC1 device (Mettler Toledo, Columbus, OH, USA). The material samples (3–10 mg) were heated under nitrogen or in air atmosphere from room temperature to 500 °C at the rate of 10 °C/min. The values of *T*_5_, *T*_25_ and *T*_50_ parameters, identified as the temperatures at which the 5, 25 and 50 wt % weight losses of the samples occurred, respectively, were determined from the TG thermograms. In turn, the *T*_max_ parameter as the maximum mass loss rate temperature was evaluated on the basis of the derived thermogravimetry (DTG) curves.

Thermogravimetry–Fourier transformed infrared spectroscopy (TG-FTIR) was performed by means of a TGA/DSC1 instrument (Mettler Toledo, Columbus, OH, USA) coupled with an FTIR Nicolet 380 spectrometer (Thermo Fisher Scientific, San Jose, CA, USA). The samples (about 10 mg) were placed in a ceramic crucible and heated from 25 to 900 °C at the rate of 10 °C/min in air atmosphere. The gas carried the decomposition products from the TG through a stainless-steel line into a gas cell with KBr crystal windows for infrared (IR) detection. Both the transfer line and the gas cell were kept at 200 °C to prevent gas condensation. IR spectra were recorded in the spectral range of 4000–500 cm^−1^ with a resolution of 4 cm^−1^ and an average number of scans of 16.

The fire performance was characterized using a mass loss cone microcalorimeter (Fire Testing Technology Ltd., East Grinstead, UK) according to the standard ISO 13927, at the external heat flux of 35 kW/m^2^. The dimensions of the samples were 100.0 mm × 100.0 mm × 2.0 mm. The specific data, including the heat release rate (HRR, kW/m^2^) and the total heat release (THR, MJ/m^2^), were collected or calculated according to the data during combustion. The typical results from the cone microcalorimeter tests were taken as the average from two measurements.

## 3. Results and Discussion

### 3.1. TG Studies

**Figure 1:**
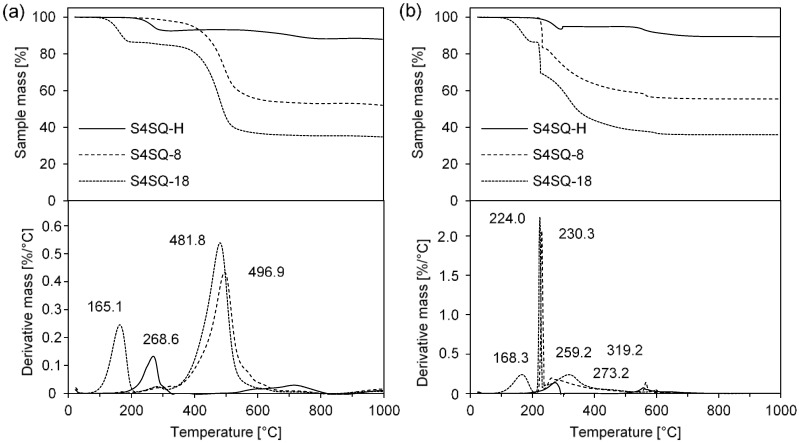
Thermogravimetry (TG)/derived thermogravimetry (DTG) curves of neat siloxane-silsesquioxane (S4SQ) resins (**a**) under nitrogen and (**b**) in air atmosphere.

**Scheme 1:**
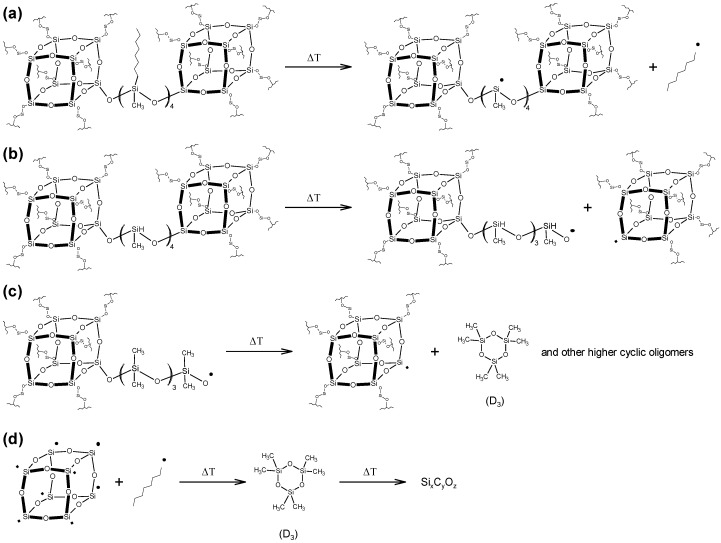
A possible mechanism for the thermal degradation of siloxane-silsesquioxane resins followed by (**a**) split-off of the alkyl substituents from the siloxane groups, (**b**) detachment of the siloxane chains from the silsesquioxane cages, (**c**) split of the siloxane chains from the resin structure and (**d**) thermal decomposition of the silsesquioxane cages to small cyclic oligomers and then to the ceramic Si_x_C_y_O_z_ structure.

Knowledge of the thermal behavior of neat siloxane-silsesquioxane resins was required to understand their role in the degradation of the PP/S4SQ composites. The TG/DTG curves of neat siloxane-silsesquioxane resins under nitrogen and in air atmosphere are shown in Figure 1. The performed analyses and the literature data [55,56,57,58] allowed us to propose a possible mechanism for the thermal degradation of the siloxane-silsesquioxane resins, as shown in Scheme 1 and discussed below.

Thermal degradation of the non-functionalized S4SQ-H resin under nitrogen was found to be a two-step process (Figure 1a). In the first step, which started at about 200 °C, detachment of the siloxane chains from the silsesquioxane cages could take place (Scheme 1b). Then, small cyclic oligomers such as hexamethylcyclotrisiloxane (D_3_) and other higher cyclic oligomers could be obtained (Scheme 1c) as a result of the split of the siloxane chains from the resin structure [55]. The small growth of the sample mass at 300 °C may be explained by the fallout of the silica derivatives from the gas phase oxidation [56]. The second step of the degradation process began at about 500 °C and was most probably associated with thermal decomposition of the silsesquioxane cages to small oligomers and then to the ceramic Si_x_C_y_O_z_ structure (Scheme 1d) [55,57].

The *n*-octadecyl-functionalized S4SQ-18 resin also degraded in a two-step process (Figure 1a). However, the first step of degradation of this compound started at much lower temperatures (~100 °C), which could be assigned to the split-off of the alkyl substituents from the siloxane groups (Scheme 1a) [58]. Then, at about 300 °C, the second step began, which might be attributed to the thermal decomposition of the resin structure to Si_x_C_y_O_z_ (Scheme 1b–d) [55,57]. Interestingly, degradation of the S4SQ-8 resin which contained the *n*-octyl groups generally occurred in one step (Figure 1a). The initial weight loss started at about 300 °C and could be associated with the loss of the organic groups (Scheme 1a) and formation of the ceramic residue (Scheme 1b–d) at the same time. It should also be noted that the S4SQ-8 resin, which contains slightly shorter *n*-alkyl substituents, exhibited higher thermal stability than the S4SQ-18 compound.

Moreover, based on the amounts of silica residues at 1000 °C (S4SQ-H: 87.9%; S4SQ-8: 52.0%; S4SQ-18: 34.8%), it is possible to assess the approximate weight fraction of the organic substituents in the functionalized resins. It turns out that the *n*-octyl and *n*-octadecyl substituents constituted about 35% and 53% of the S4SQ-8 and S4SQ-18 resins respectively.

The thermo-oxidative degradation of the siloxane-silsesquioxane resins (Figure 1b) gave similar results to those obtained in the analyses performed under nitrogen (Figure 1a). However, the oxidizing action of the oxygen molecules accelerated the degradation processes [59,60,61], which started at much lower temperatures.

Degradation of the non-functionalized S4SQ-H resin in air atmosphere was also a two-step process (Figure 1b). The siloxane chains started to be detached from the silsesquioxane cages at about 220 °C (Scheme 1b), after which they could be transformed into the oligomers such as D_3_ (Scheme 1c) [55]. The silsesquioxane cages, which are the most thermally stable part of the siloxane-silsesquioxane resin structure, were decomposed at about 500 °C, probably to the ceramic residue (Scheme 1d) [55,57]. Once again, a small growth of the sample mass was observed at about 300 °C, which could be assigned (1) to the thermal oxidation of Si-H bonds and formation of Si-O-Si bonds [62] and/or (2) to the fallout of the silica derivatives from the gas phase oxidation [56].

In the case of the siloxane-silsesquioxane resin functionalized with the *n*-octadecyl groups (S4SQ-18), its thermal degradation began at about 100 °C (Figure 1b). The explanation for this may be the split-off of the long *n*-alkyl chain substituents in the resin structure by peroxidation and subsequent fragmentation through classical radical pathways (Scheme 1a) [21,58,61]. Then, at about 220 °C, the organic groups were probably suddenly lost and thermal degradation of the resin structure began (Scheme 1b,c). Next, the sample mass decreased gradually while the temperature rose to 600 °C, at which the ceramic Si_x_C_y_O_z_ residue could be formed (Scheme 1d) [55,57]. As regards thermal degradation of the S4SQ-8 resin, it started at about 220 °C with a sharp weight loss, which was assigned to the simultaneous sudden loss of the *n*-octyl groups (Scheme 1a) and formation of the ceramic residue (Scheme 1b–d).

The silica residues at 1000 °C (S4SQ-H: 89.3%; S4SQ-8: 55.4%; S4SQ-18: 35.9%) indicate that the approximate weight fractions of the alkyl groups in the S4SQ-8 and S4SQ-18 resins were 34% and 53%, respectively. These values are consistent with the amount of silica residues from the analysis performed under nitrogen. It may, hence, be concluded that the presence of oxygen accelerated thermal decomposition of the siloxane-silsesquioxane resins, especially the split-off of the alkyl substituents from the siloxane groups, but it did not influence the amounts and the composition of the residues.

**Figure 2:**
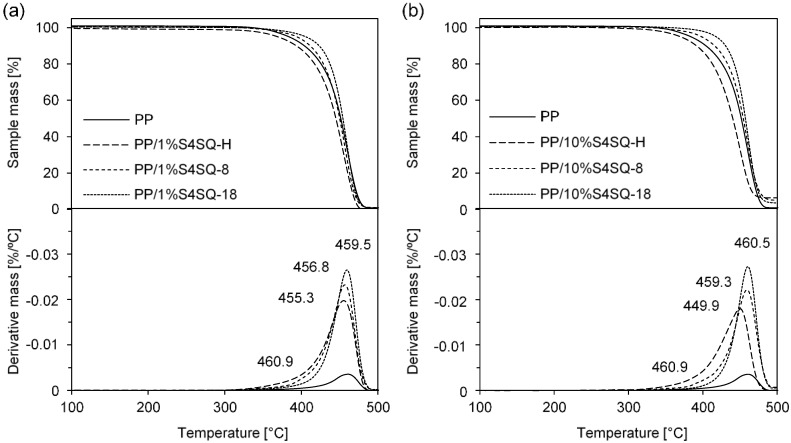
TG/DTG curves of neat PP as well as (**a**) PP/1%S4SQ and (**b**) PP/10%S4SQ composites (under nitrogen atmosphere).

Figure 2 shows the TG/DTG curves recorded under nitrogen for neat PP and PP composites filled with 1 and 10 wt % of the S4SQ fillers. All these materials presented similar one-step degradation shapes of the TG curves, which means that siloxane-silsesquioxane resins did not change the degradation mechanism of neat PP and that these fillers could only accelerate or delay the onset of this process (Figure 2).

The results of the TG analyses performed under nitrogen and in air atmosphere for neat PP and PP composites containing S4SQ fillers are shown in Table 1.

In the case of the studies carried out under nitrogen, the addition of the siloxane-silsesquioxane resins into the PP matrix caused a slight decrease of the *T*_max_ values in comparison with neat PP, irrespective of the kind of resin used (Figure 2, Table 1). The filler contents had a negligible influence on the values of this parameter.

The *T*_5_, *T*_25_ and *T*_50_ values were also found to decrease after the introduction of 1 wt % of S4SQ-H resin into polypropylene (Table 1). Increasing the wt % content of this filler in the material resulted in a slight decrease in the values of these parameters. However, in the presence of 1 wt % of S4SQ-8 and S4SQ-18 fillers, a significant increase in the *T*_5_, *T*_25_ and *T*_50_ values was demonstrated (Table 1). Moreover, the values of these parameters increased with the increasing filler wt % content in the PP matrix. This could be explained by good compatibility between the PP chains and resins containing *n*-octyl and *n*-octadecyl substituents in their structures which contributed to a more uniform dispersion of S4SQ-8 and S4SQ-18 particles in the PP matrix. Similar observations were reported in our previous work [32] in the case of polypropylene composites containing POSS nanofillers with *n*-octyl and *n*-octadecyl groups attached to the silicon-oxygen cages.

**Figure 3:**
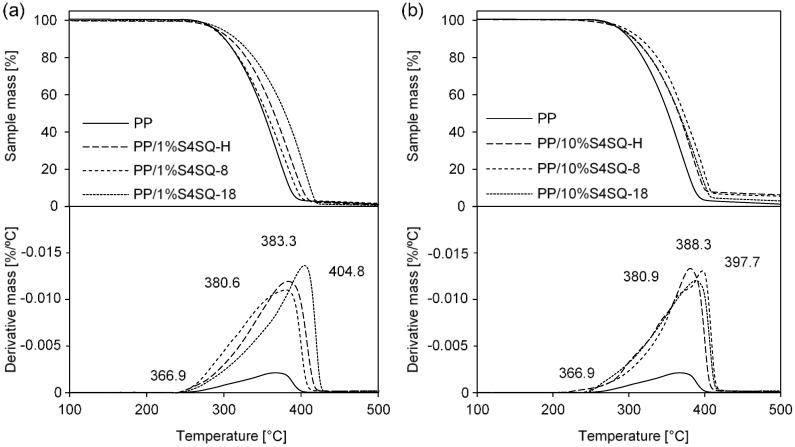
TG/DTG curves of neat PP as well as (**a**) PP/1%S4SQ and (**b**) PP/10%S4SQ composites (in air atmosphere).

As regards the results of the TG analyses carried out in air atmosphere, the *T*_max_ values were increased by 20–40 °C after incorporation of the siloxane-silsesquioxane resins to the PP matrix (Figure 3, Table 1). Increasing the content of these fillers in the materials above 5 wt % resulted in the slight lowering of *T*_max_, which is probably associated with the formation of the filler aggregates in the composites. These results are in accordance with the literature data [15,32,33,63,64]. The highest improvement of the *T*_max_ parameter was observed for the samples containing resin functionalized with the *n*-octadecyl substituents (Table 1).

In the case of the PP/S4SQ-H composites with 1 and 5 wt % filler contents, *T*_5_ values increased by about 2–3 °C, and the *T*_25_ and *T*_50_ values by more than 10 °C, in comparison with neat PP (Table 1). However, any further increase of the S4SQ-H content (up to 10 wt %) worsened the thermal stability of the PP/S4SQ-H composites.

A considerable improvement in *T*_5_, *T*_25_ and *T*_50_ was observed for composites with the functionalized S4SQ-8 and S4SQ-18 resins, in comparison with neat PP (Table 1), irrespective of the S4SQ filler content. However, it should be noted that the highest thermal stability for the PP/S4SQ-8 and PP/S4SQ-18 composites was observed for the samples containing 5 and 1 wt % of resins, respectively.

The high thermal stability of polypropylene composites with siloxane-silsesquioxane resins as fillers could be explained by the possible formation of a ceramic layer on the surface of the composite material during thermal degradation of S4SQ. As a result, the heat flux to the sample, oxygen diffusion towards the bulk material, and the evolution of the volatile degradation gases from the sample were significantly limited, as was also demonstrated for POSS-containing composites based on polypropylene, polyethylene and polystyrene matrices [4,16,63].

Moreover, some correlation between the thermal stability of the composites and S4SQ filler dispersion in the polymer matrix was observed. The filler aggregates may cause some loosening of the polymer chain packing, as well as some increase in the free volumes which would facilitate the access of oxygen to the material. Thus, uniform dispersion of the siloxane-silsesquioxane resins in PP, which was achieved at lower filler loadings, led to the improved thermal stability of the obtained materials. However, as the siloxane-silsesquioxane resins tend to aggregate at higher wt % contents, the thermal stability of the composites was decreased.

### 3.2. TG-FTIR Studies

**Figure 4:**
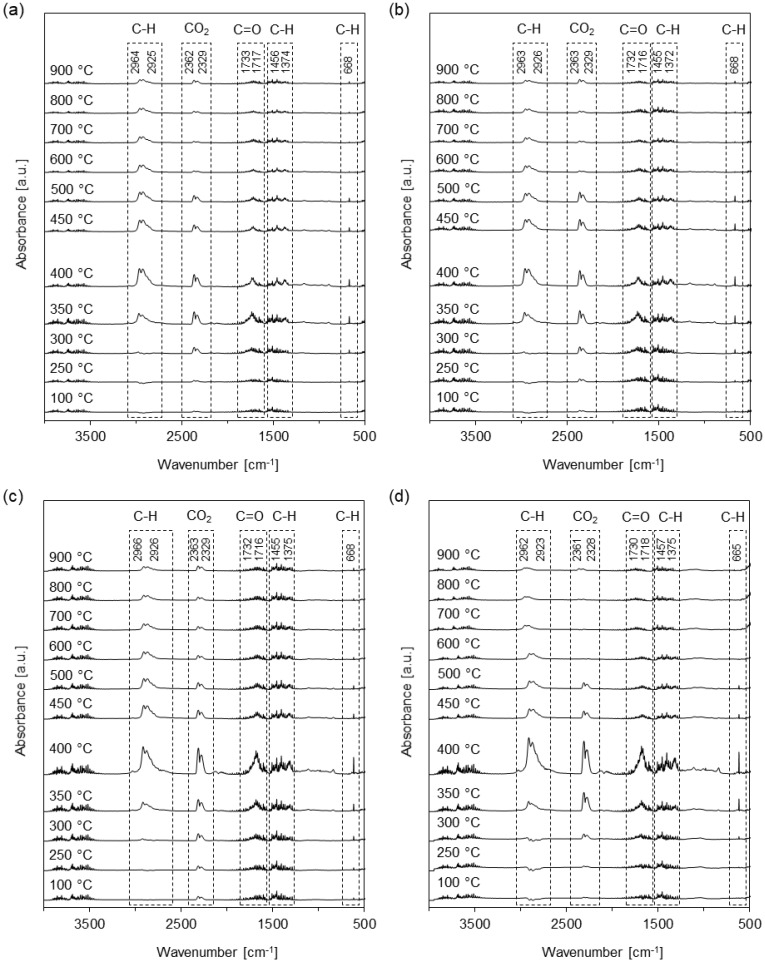
2D thermogravimetry-Fourier transform infrared spectrometry (TG-FTIR) spectra of pyrolysis products of (**a**) neat PP, (**b**) PP/1%S4SQ-H, (**c**) PP/1%S4SQ-8 and (**d**) PP/1%S4SQ-18 at various temperatures.

In order to evaluate comprehensively the influence of the siloxane-silsesquioxane resins on the thermal stability of PP/S4SQ composites, the gaseous pyrolysis products of neat PP and composites containing 1 wt % of filler were analyzed by the TG-FTIR method. The detailed characteristics of each TG-FTIR spectrum obtained at different temperatures are shown in Figure 4.

Thermal decomposition of both neat PP (Figure 4a) and PP/S4SQ composites (Figure 4b–d) started at about 300 °C. The main degradation products were alkenes, dienes and alkanes, as the absorbances at about 2964 (assigned to the stretching, asymmetric vibrations of -CH_3_ groups), 2925 (stretching, asymmetric vibrations of -CH_2_- groups), 1456 (deformation, asymmetric vibrations of -CH_3_ groups), 1374 (deformation, asymmetric vibrations of -CH_3_ groups) and 668 cm^−1^ (out of plane deformation vibrations of =CH- groups) were clearly visible at 300–600 °C. Moreover, sharp signals in the range of 1600–1800 cm^−1^ may be attributed to carbonyl-containing compounds such as aldehydes (1733 cm^−1^), ketones (1717 cm^−1^) and carboxylic acids (1705 cm^−1^). Furthermore, the release of nonflammable gases such as H_2_O, CO_2_ and CO could also be demonstrated by the signals at 3400–4000, 2300–2390 and 2080–2180 cm^−1^, respectively.

**Figure 5:**
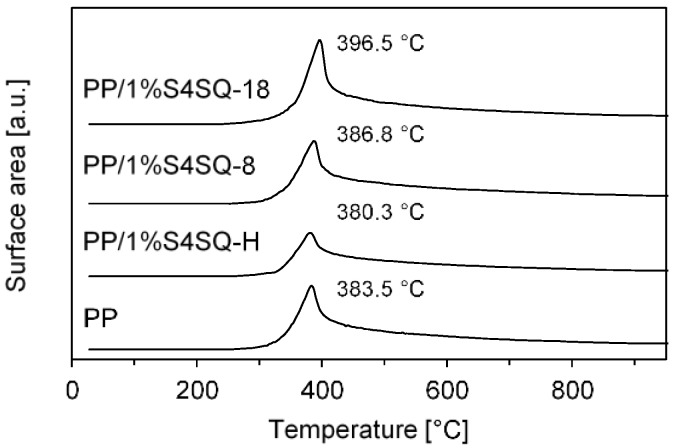
The evolution curves of the hydrocarbons (in the range of 2800–3000 cm^−1^) evolved from neat PP and PP/S4SQ composites vs. temperature.

No additional signals which might be associated with thermal degradation of siloxane-silsesquioxane resins were observed in the spectra of the PP/S4SQ composites (Figure 4b–d), in comparison with the spectrum of neat PP (Figure 4a). It should be noted that the main differences between the spectra of neat PP and the PP/S4SQ composites were visible only at the intensities of the absorbance peaks. A greater increase in the intensity of the peaks associated with the hydrocarbons (in the range of 2800–3000 cm^−1^), which are the main products of PP decomposition, was found in the case of the PP/1%S4SQ-8 (Figure 4c) and PP/1%S4SQ 18 (Figure 4d) composites. Figure 5 presents the evolution curves of hydrocarbons from the analyzed samples, which are shown as the surface area of the peaks in the range of 2800–3000 cm^−1^ versus temperature.

The initial release of hydrocarbon gases for neat PP and PP/S4SQ composites occurred at almost the same temperature (at about 260 °C) (Figure 5). However, the maximum of the signal for the PP/1%S4SQ-18 composite appeared at 396.5 °C and it was about 10 °C higher than that for neat PP (383.5 °C), as well as for PP/1%S4SQ-H (380.3 °C) and for PP/1%S4SQ-8 (386.8 °C). Moreover, a greater increase in the intensity of the hydrocarbons in the case of the materials with the functionalized S4SQ-8 and S4SQ-18 resins was probably caused by the presence in the gaseous products of the pyrolysis of both the PP matrix and *n*-alkyl substituents which were split off from the siloxane groups of the filler molecules.

**Scheme 2:**
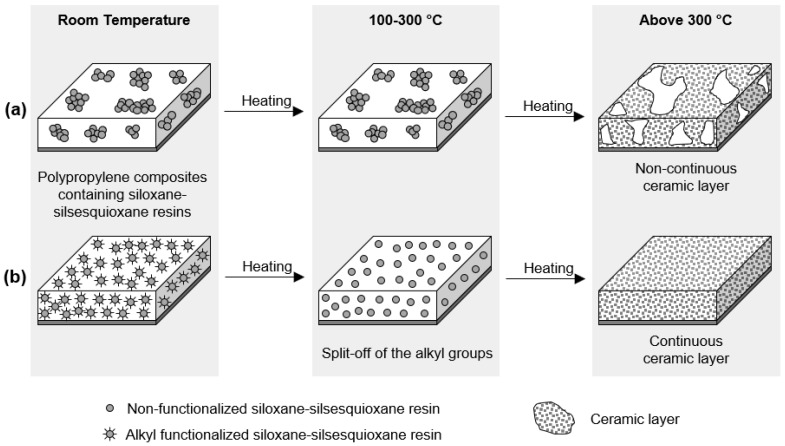
The possible roles of (**a**) non-functionalized and (**b**) *n*-alkyl-functionalized siloxane-silsesquioxane resins during thermal degradation of PP/S4SQ composites.

On the basis of the performed analysis, the possible role of the siloxane-silsesquioxane resins during the PP matrix decomposition may be explained. Firstly, decomposition of all the analyzed materials started at almost the same time because the release of the gaseous degradation products occurred at similar temperatures. Thus, it might be assumed that the applied siloxane-silsesquioxane resins did not significantly delay the thermal decomposition of polypropylene. Moreover, it seems that the S4SQ resins do not quench the free radicals generated during decomposition of the PP matrix. Secondly, the highest growth of the intensity of the hydrocarbons occurred in the PP/1%S4SQ-8 and PP/1%S4SQ-18 composites proved that the *n*-alkyl substituents in the siloxane groups of the filler particles did not improve the thermal stability of those composites. However, those groups had a crucial role in the uniform dispersion of the siloxane-silsesquioxane resins in the PP matrix, as it is shown in Scheme 2. Although they are detached from the filler particles at higher temperatures, they provide the formation of a continuous thin ceramic layer on the specimen surface during its thermal decomposition or combustion (Scheme 2b). This layer represents an effective physical barrier which slows down the decomposition of the polymer matrix. On the other hand, non-functionalized siloxane-silsesquioxane resin forms a non-continuous ceramic layer on the sample surface during the composite’s decomposition, which is not an effective barrier (Scheme 2a). The formation of the solid ceramic layer on the sample surface could also be proved by the lack of any signals associated with gaseous products of the thermal degradation of the siloxane-silsesquioxane resins (besides the alkyl substituents).

### 3.3. Fire Behavior

Neat PP and the PP/S4SQ composite samples were subjected to cone calorimeter investigations which gave comprehensive information on their performance in a rather well-defined fire test scenario. The main parameters obtained from the cone calorimeter measurements, such as time to ignition (TTI), heat release rate (HRR), fire performance index (FPI), total heat released (THR), effective heat of combustion (EHC), maximum average rate of heat emission (MAHRE) and mass loss during combustion, are reported in Table 2.

**Figure 6:**
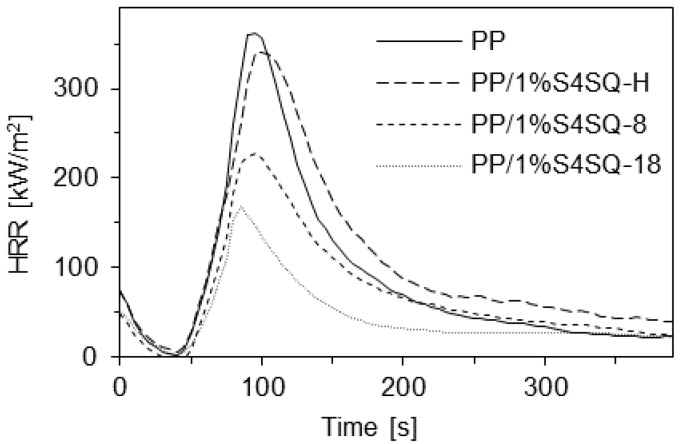
Dynamic curves of HRR versus time for neat PP and PP/S4SQ composites.

The addition of just 1 wt % of S4SQ-H resin into the PP matrix did not change the time to ignition (TTI) value in comparison with neat PP (Figure 6). However, increasing the content of the filler in the material resulted in significantly lowered TTI values. On the other hand, the values of that parameter for the PP/S4SQ-8 and PP/S4SQ-18 composites were equal or even higher with respect to neat PP. Thus, it could be concluded that the addition of the S4SQ-H filler into the PP matrix accelerated the combustion process, while the S4SQ-8 and S4SQ-18 particles slowed it down.

The peak of the heat release rate (PkHRR) is a characteristic that displays a very strong and quite complex dependence on the fire scenario [65]. A very sharp HRR peak of neat PP appears at the range of 80–100 s, with a PkHRR of 364 kW/m^2^ (Figure 6). The addition of just 1 wt % of the S4SQ resins into the PP matrix caused a significant reduction of PkHRR in comparison with neat PP. It should be noted that all the PP/1%S4SQ composites showed a peak in the heat release rate after the initial strong increase in the heat release rate, as shown in Figure 6, which proves the formation of a protective surface layer [66]. It may be assumed that a char layer is formed during the combustion process on the sample surface and siloxane-silsesquioxane particles undergo a series of oxidation reactions to form Si_x_C_y_O_z_ residue. This enhanced char structure protects and insulates the underlying polymer from any further degradation [3]. Similar results were observed by other authors in the case of composites containing POSS fillers [4,66]. Interestingly, increasing the amount of the applied S4SQ fillers in the materials generally resulted in the growth of the PkHRR values. The explanation of this effect may be an aggregation of the siloxane-silsesquioxane resin particles in the polymer which caused the formation of a non-continuous thin ceramic layer on the specimen surface during combustion. This layer does not provide an effective physical barrier for the combustion processes (Scheme 2). Furthermore, a greater deteriorating effect on the flammability properties of PP was found in the case of the composites with S4SQ-8 and S4SQ-18 resins in comparison with S4SQ-H. It may be concluded that the presence of the long *n*-alkyl chain substituents in the structure of the siloxane-silsesquioxane resins had a favorable effect on the filler dispersion in the PP matrix and consequently on the limitation of composite combustion.

The fire performance index (FPI) is used to predict whether a material can easily develop drastic combustion after ignition. FPI is defined as the ratio of TTI to PkHRR, and it indicates the fire resistance of the analyzed material [67]. It can be seen from Table 2 that the PP/1%S4SQ-8 and PP/1%S4SQ-18 composites have the highest FPI values among the all the analyzed samples; hence, they are characterized by the best fire resistance. On the other hand, in the case of the PP/5%S4SQ-H and PP/10%S4SQ-H materials, the FPI values were more than two times lower than for neat PP, which indicates their higher susceptibility to combustion.

**Figure 7:**
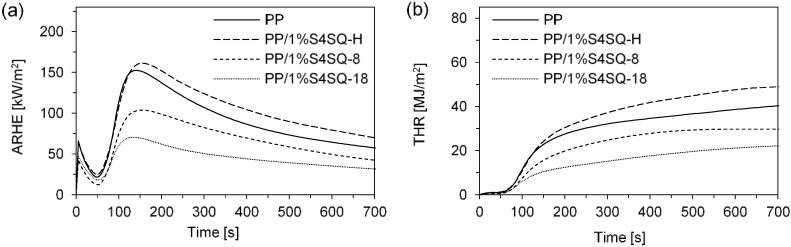
Heat evolving during combustion for neat PP and PP/S4SQ composites: (**a**) average rate of heat emission (AHRE) and (**b**) total heat released (THR).

The maximum average rate of heat emission (MAHRE) is a good measure of the propensity for fire development under real scale conditions [68]. MAHRE for the PP/1%S4SQ-8 and PP/1%S4SQ-18 composites shows a significant reduction by 25% and 56%, respectively, in comparison with neat PP (Figure 7a). However, increasing the wt % content of S4SQ-8 and S4SQ-18 fillers in the PP matrix or the addition of S4SQ-H filler resulted in a clear growth of the MAHRE values. Therefore, small amounts of the functionalized siloxane-silsesquioxane resins have favorable effects on flame retardancy in the PP/S4SQ composites, whereas a high wt % content of these fillers deteriorates the flame-retardant properties of the obtained materials.

Furthermore, the amount of the non-combustible fraction in the materials may be determined on the basis of the total heat release (THR) values. Once again, a decrease in THR was observed in the case of the PP/1%S4SQ-8 and PP/1%S4SQ-18 composites compared with neat PP (Figure 7b). A lower THR value may indicate that a part of the polymer is not completely combusted but that it could undergo a carbonization process. On the other hand, in the case of the PP/S4SQ-H composites, as well as the PP/S4SQ-8 and PP/S4SQ-18 with 5 and 10 wt % filler contents, the THR values were higher in comparison with neat PP.

The values of the effective heat of combustion (EHC) of all the analyzed PP/S4SQ composites were equal to or lower than that for neat PP. Hence, the combustion kinetics of these materials were affected by the presence of the siloxane-silsesquioxane resins. This is generally in agreement with the changes in the PkHRR values.

**Figure 8:**
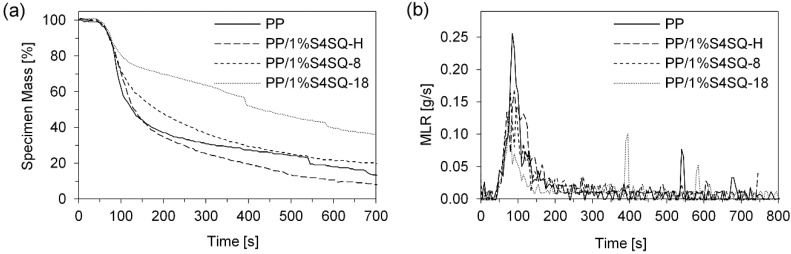
Mass curves during combustion for neat PP and PP/S4SQ composites: (**a**) sample mass (normalized on initial weight) and (**b**) mass loss rate (MLR).

The mass loss values were found to be reduced for the PP/1%S4SQ-8 and PP/1%S4SQ-18 composites in comparison with neat PP (Table 2). The changes in this parameter for the other PP/S4SQ composites were not significant. Moreover, the addition of 1 wt % of the S4SQ-8 and S4SQ-18 resins into the PP matrix resulted in a decrease in the mass loss rate, as shown in Figure 8. The lowest rate of combustion may be attributed to the homogeneous dispersion of these fillers in the matrix. However, increasing the wt % content of the filler in the materials resulted in limiting this effect.

## 4. Conclusions

The influence of a novel group of silsesquioxane derivatives, siloxane-silsesquioxane resins, on the thermal stability and combustion process of polypropylene composites was examined for the first time. The thermogravimetric studies of neat, non-functionalized, *n*-octyl- and *n*-octadecyl-functionalized S4SQ resins revealed complicated processes in their decomposition which were strongly dependent on the structures of the resins. Based on these results, a possible mechanism for the thermal degradation of the siloxane-silsesquioxane resins was proposed. The TG analyses of the PP materials demonstrated that the S4SQ resins may improve the thermal stability of the composites, especially when they are homogeneously dispersed in the PP matrix. The role of the siloxane-silsesquioxane resins during the thermal decomposition of PP/S4SQ composites was comprehensively evaluated using TG-FTIR. It was found that the presence of the alkyl substituents in the resin structure improved the material’s homogeneity, but they were split off at higher temperatures. However, the obtained uniform dispersion of the filler particles in the polymer matrix resulted in the formation of a continuous ceramic layer on the sample surface. Finally, the possible flame retardancy effect of the siloxane-silsesquioxane resins on the PP matrix was examined by cone calorimeter tests. It turns out that small amounts of *n*-alkyl-functionalized siloxane-silsesquioxane resins effectively slow down the combustion process of the material. The values of the PkHRR, MAHRE, THR and EHC parameters were significantly decreased after addition of just 1 wt % of S4SQ-8 and S4SQ-18 filler particles into the PP.

## Figures and Tables

**Table 1:** TG data of neat polypropylene (PP) and PP/S4SQ composites.

Sample	Nitrogen Atmosphere				Air Atmosphere			
	*T*_max_ (°C)	*T*_5_ (°C)	*T*_25_ (°C)	*T*_50_ (°C)	*T*_max_ (°C)	*T*_5_ (°C)	*T*_25_ (°C)	*T*_50_ (°C)
neat PP	460.9	383.7	432.6	451.5	366.9	289.1	325.4	351.8
PP/1%S4SQ-H	455.3	369.1	424.5	445.2	383.3	290.9	336.8	365.3
PP/5%S4SQ-H	-	-	-	-	382.5	292.0	336.5	365.5
PP/10%S4SQ-H	449.9	370.7	419.8	440.7	380.9	282.5	332.6	363.9
PP/1%S4SQ-8	456.8	394.1	434,2	450.0	380.6	291.6	326.8	355.7
PP/5%S4SQ-8	-	-	-	-	400.6	301.5	350.7	380.2
PP/10%S4SQ-8	459.3	398.8	439.0	454.8	388.3	297.8	345.5	372.6
PP/1%S4SQ-18	459.6	405.7	441.5	454.2	404.8	298.4	347.6	379.6
PP/5%S4SQ-18	-	-	-	-	409.0	298.0	346.0	376.7
PP/10%S4SQ-18	460.5	417.7	445.6	457.0	397.7	290.3	336.2	366.2

**Table 2:** Main parameters obtained from cone calorimeter measurements for neat PP and PP/S4SQ composites.

Sample	TTI(s)	PkHRR (kW/m^2^)	FPI (m^2^·s/kW)	MAHRE (kW/m^2^)	THR (MJ/m^2^)	EHC (MJ/kg)	Mass Loss (g)
PP	40	364	0.110	154	40	76	15.6
PP/1%S4SQ-H	41	354	0.116	185	47	74	16.7
PP/5%S4SQ-H	21	500	0.042	223	44	77	15.9
PP/10%S4SQ-H	19	445	0.043	214	44	78	16.6
PP/1%S4SQ-8	43	227	0.190	115	29	59	14.3
PP/5%S4SQ-8	40	481	0.083	215	48	75	17.0
PP/10%S4SQ-8	-	-	-	-	-	-	-
PP/1%S4SQ-18	40	168	0.247	68	22	66	12.3
PP/5%S4SQ-18	43	328	0.131	160	42	78	15.6
PP/10%S4SQ-18	47	391	0.120	183	47	51	16.2
